# Spt3 and Spt8 Are Involved in the Formation of a Silencing Boundary by Interacting with TATA-Binding Protein

**DOI:** 10.3390/biom13040619

**Published:** 2023-03-30

**Authors:** Kazuma Kamata, Takahito Ayano, Masaya Oki

**Affiliations:** 1 Department of Applied Chemistry & Biotechnology, Graduate School of Engineering, University of Fukui, Fukui 910-8507, Japan; kazuma_kjp@yahoo.co.jp (K.K.);; 2 Japan Society for the Promotion of Science for Young Scientists (JSPS), Tokyo 102-0083, Japan; 3 Life Science Innovation Center, University of Fukui, Fukui 910-8507, Japan

**Keywords:** boundary, SAGA complex, TBP, subtelomere, gene silencing, *Saccharomyces cerevisiae*

## Abstract

In *Saccharomyces cerevisiae*, a heterochromatin-like chromatin structure called the silencing region is present at the telomere as a complex of Sir2, Sir3, and Sir4. Although spreading of the silencing region is blocked by histone acetylase-mediated boundary formation, the details of the factors and mechanisms involved in the spread and formation of the boundary at each telomere are unknown. Here, we show that Spt3 and Spt8 block the spread of the silencing regions. Spt3 and Spt8 are members of the Spt-Ada-Gcn5-acetyltransferase (SAGA) complex, which has histone acetyltransferase activity. We performed microarray analysis of the transcriptome of *spt3Δ* and *spt8Δ* strains and RT-qPCR analysis of the transcript levels of genes from the subtelomeric region in mutants in which the interaction of Spt3 with TATA-binding protein (TBP) is altered. The results not only indicated that both Spt3 and Spt8 are involved in TBP-mediated boundary formation on the right arm of chromosome III, but also that boundary formation in this region is DNA sequence independent. Although both Spt3 and Spt8 interact with TBP, Spt3 had a greater effect on genome-wide transcription. Mutant analysis showed that the interaction between Spt3 and TBP plays an important role in the boundary formation.

## 1. Introduction

Transcriptional silencing is determined by histone modifications in *Saccharomyces cerevisiae*. The Sir2/4 proteins are recruited to the target region and deacetylate the neighboring histone H4 at Lysine 16. Sir3 interacts with deacetylated histones, followed by interaction with Sir2/4 proteins, leading to further histone deacetylation by Sir2 [1,2,3,4]. Thus, the silencing region formed by Sir proteins is expanded by this “step-wise” mechanism. Transcription of the *HM* regions, which is the sex-determining region, and the genes of telomeres are silenced by the Sir2/3/4 complex. On the other hand, *rDNA* transcription is silenced by Sir2 [5,6,7]. The histone acetyltransferase Sas2 is the counterpart of Sir2, a NAD+-dependent histone deacetylase [8,9], and Sas2 mediates the establishment of heterochromatin boundaries. The different mechanism of boundary formation from that of Sas2 histone modifications has been reported for the *tDNA* region adjacent to the *HMR* silencing region and for the promoter of the *CHA1* gene near the *HML* silencing region, which functions as an insulator [10,11,12,13]. However, many questions remain, such as the mechanism of boundary formation in native configurations, the number of boundary-forming factors, and the genetic regions regulated by boundary fluctuations.

Boundary factors in *Saccharomyces cerevisiae* have been identified by screening with the *HMR* locus and are roughly divided into eight groups: histone modification, transcriptional factor, cell cycle, SWI/SNF, TFIID, mediator, others, and unknown function [14]. In more detail, some of the components of the Spt-Ada-Gcn5-acetyltransferase (SAGA) complex were included in the histone modification group. The SAGA complex consists of 19 proteins, including a histone acetyltransferase and a H2B deubiquitinating enzyme, which are known to promote transcription initiation by interacting with TBP [15,16,17,18,19,20,21,22,23]. We previously focused on the large number of components of the SAGA complex uncovered earlier during screening, and reported that Sgf73, which has deubiquitination activity, Sgf29, which is involved in the recognition of H3K9me2 and H3K9me3, and Ada1, the core component of the SAGA complex, are important for boundary formation [24,25,26]. Screening has also revealed that TBP1 (also known as Spt15), which is known to interact directly with SAGA complex components Spt3 and Spt8, is capable of boundary formation; however, it is still not understood how the SAGA complex and TBP1 co-operate in boundary formation.

Spt3 and Spt8 are components of the histone modification complex SAGA [15,27]. Both Spt3 and Spt8 interact with TATA-binding proteins (TBPs) and regulate target gene expression positively and negatively [28,29,30,31,32,33]. As an example of negative regulation, binding of Spt3 and Spt8 to TBP prevents TBP from binding to the TATA box, resulting in repression of *HIS3* and *TRP3* genes [34,35]. However, when the binding of Spt3 and Spt8 to TBP is weakened, TBP binds to the TATA box and transcription is promoted [30]. In another mechanism, Spt3 interacts with Gcn5 and inhibits its function. Gcn5 is phosphorylated and activated by Snf1, but Spt3 inhibits Gcn5 function by binding to Gcn5 at the Snf1 interaction site [33]. Others have reported that deletion of *SPT3* gene decreases transcription of genes encoding a- and α-factors and causes defects in mating [36]. In addition, Spt3 is a component of both the SAGA complex and a SAGA-like (SLIK) complex, with a similar structure and function to the SAGA complex; however, the SLIK complex does not contain Spt8 [36,37,38,39]. The SLIK complex contains a C-terminal truncated Spt7 (1–1141 amino acids) instead of full-length Spt7 [40].

In this study, we found that Spt3 and Spt8 contribute to the formation of a silencing boundary at the right subtelomere of chromosome III (Chr III), and that the boundary function depends on the intensity of the interaction between the SAGA complex and TBPs via Spt3 and Spt8.

## 2. Materials and Methods

### 2.1. Yeast Strains

The *KanMX* gene was used to construct strains with deletion of the required gene (Knockout Strain Collection, Open Biosystems). To construct the deletion strain, a *KanMX* cassette flanked by the promoter and terminator of the target genes was prepared by PCR and introduced into yeast cells [41,42]. Transformed cells were selected using medium containing G418. *SIR3* deletion strains by *HphMX* were constructed using the same method used to construct *KanMX* strains and selected by Hygromycin B.

To obtain amino acid residue substitution mutant strains, the particular target gene was first replaced with the *URA3* gene and transformants were selected on uracil-free synthetic complete (SC) medium. Subsequently, *URA3* was replaced with the amino acid residue substituted target gene and the required colonies were selected on 5-fluoroorotic acid (5-FOA) medium [43]. Strains with the substituted telomeric sequence were constructed using the same method used to construct amino acid residue substitution mutant strains.

For *3xFlag-SPT7* strain construction, first, the plasmid (*URA3*-*SPT7* promoter-3xFlag-*SPT7* ORF) was inserted in the *SPT7* ORF, and transformants (*SPT7* promoter-*SPT7* ORF- *URA3*-*SPT7* promoter-3xFlag-*SPT7* ORF) were selected on uracil-free SC medium. Subsequently, *URA3* was deleted by intrachromosomal homologous recombination of the *SPT7* promoter and the *3xFlag-SPT7* strain was selected on 5-FOA medium. Other strains producing Flag-tagged proteins were constructed using the same methods.

G418 for *KanMX* selection and Hygromycin B for *HphMX* selection were added to YPD, uracil-free SC medium for *URA3* selection, and 5-FOA medium for *URA3* counterselection were used as needed.

Yeast cultures were grown on YPD medium at 30 °C.

The strains used in this study are listed in Table 1.

### 2.2. Plasmids

The plasmids bearing target genes fused to a Flag-tag encoding sequence were constructed by amplification of target genes with Gflex DNA polymerase (TaKaRa, Shiga, Japan) by PCR. The PCR products were purified using the GEX™ PCR DNA and Gel Purification Kits (GE Healthcare, Chicago, IL, USA) and inserted into pRS406 [44].

The *Escherichia coli* strain DH5α was used as a host for the plasmids, which were cultured in LB medium containing ampicillin at 37 °C.

### 2.3. RNA Extraction and Real-Time PCR Analysis

RNA analysis was performed as described previously [26]. The primers used for real-time PCR are listed in Table 2.

### 2.4. Microarray Analysis

Yeast cells were grown in YPD medium to mid-log phase and were collected by centrifugation. Total RNA was extracted from whole cell lysates using hot phenol and RNA (200 ng) was used for first strand cDNA synthesis using the GeneChip^®^ 3′ IVT Express Kit (Affymetrix, Santa Clara, CA, USA). aRNA (7.5 μg) was hybridized for 16 hr at 45 °C in a GeneChip Yeast Genome 2.0 Array. GeneChips were washed and stained in a Affymetrix Fluidics Station 450, and then scanned using the GeneChip Scanner 3000 7G System. The data were analyzed using Microarray Suite version 5.0 (MAS 5.0) with Affymetrix default analysis settings.

The accession number of the microarray data is GSE220290.

### 2.5. Immunoblotting

Western blotting was performed as described previously [25]. Anti-Flag M2 (Sigma, Sofia, Bulgaria) was used at a dilution of 1/5000.

### 2.6. Chromatin Immunoprecipitation (ChIP)

ChIP was performed to determine the boundary position of the Chromatin III right telomere region. ChIP assays were performed as described previously [45,46]. Yeast cells were grown until reaching OD600 = 1.5 at 30 °C in YPD medium and collected by centrifugation at 4 °C for 5 min. Cells were resuspended in 200 mL cross-linking buffer (10 mM Hepes-KOH pH 7.5, 3 mM MgCl) + 1% HCHO. Rabbit polyclonal anti-Sir3 antibody (a gift from Dr. Kamakaka) was used for immunoprecipitation. The amount of immunoprecipitated DNA was measured using the Applied Biosystems SYBR Green RT-PCR system. Chromatin VI right telomere regions were used as controls. The primers used for ChIP are listed in Table 3.

## 3. Results

### 3.1. Spt3 and Spt8 Regulate Subtelomere Genes

**Figure 1:**
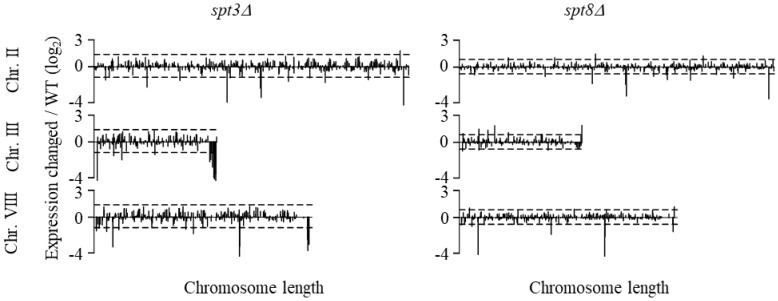
Spt3 and Spt8 regulate subtelomere gene expression. Changes in expression of genes from Chr II, III, and VIII in the *spt3Δ* and *spt8Δ* and the wild type (WT) strains as determined by microarray. Dotted lines show the average ± 2· standard deviation.

In budding yeast, the process of silencing regulation is not clear, and it is not known which regions and genes are regulated by the interaction of the SAGA complex and TBP. Microarray analysis was performed on *SPT3* deletion strain (*spt3Δ*) and *SPT8* deletion strain (*spt8Δ*) to determine which regions on the chromosome are regulated by SAGA-TBP. The *spt3Δ* strain and *spt8Δ* strain showed significant changes in subtelomere silencing compared with the wild type strain (WT) (Figure 1). The genes showing differences in expression were almost identical between *spt3Δ* and *spt8Δ* strains, but overall, expression changes were greater in *spt3Δ* than in *spt8Δ*.

**Figure 2:**
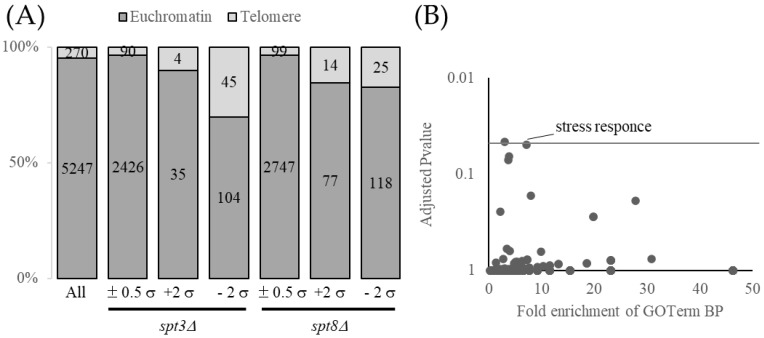
The *spt3Δ* and *spt8Δ* mutants showed a deficit in the silencing boundary. (**A**) A 100% stacked column chart of microarray analysis of the transcriptome of *spt3Δ* strain and *spt8Δ* strain. The numbers in the bar plot represent gene numbers. σ indicates standard deviation. ±0.5σ, +2σ, and −2σ indicate unchanged, upregulated, and downregulated genes, respectively, compared to WT. (**B**) The results of DAVID analysis of downregulated genes in *spt3Δ*. The GOTerm biological process (BP) category results were plotted on the *x*-axis. Fold enrichment means the ratio of the actual number of downregulated genes to the expected number of genes belonging to each term. Adjusted *p*-values were calculated by the Benjamini method.

To confirm that Spt3 and Spt8 affect subtelomeric region dominance, we analyzed trends in genes with fluctuating expression levels, focusing on their distance from telomeres. We checked the position of the genes at the chromosomes and found that 270 genes of all yeast genes (5517 genes) were located within 30 Kb from telomeres (subtelomeres), and the frequency rate of subtelomere genes was approximately 5% (270/5517). If the effects of Spt3 and Spt8 occur randomly throughout the genome, we considered that the frequency rate of affected genes in subtelomeres is about 5% in *spt3Δ* or *spt8Δ* compared with WT. In the *spt3Δ* compared with WT, there were 149 genes downregulated by a less than two-fold standard deviation (−2σ, 0.46-fold), of which 46 genes were located in the subtelomere, and the frequency rate was approximately 30% (46/149) (Figure 2A, left; *p* = 3.9 × 10^−9^, Fisher’s test). Similarly, in the *spt8Δ*, 143 genes were downregulated by <−2σ (0.59-fold) and 25 genes were located in the subtelomere. From this, the frequency rate was approximately 17% (25/143) (Figure 2A, right; *p* = 1.1 × 10^−3^). Furthermore, gene ontology analysis of the downregulated genes in *spt3Δ* showed enrichment of genes involved in the stress response (Figure 2B). This is consistent with previous reports showing that, among TBP target genes, SAGA-dominated genes are involved in the stress response [32], and that many stress response genes are contained in subtelomeric region [47,48]. These results indicate that the genes regulated by the SAGA-TBP interaction are strongly enriched in subtelomeric regions.

### 3.2. Spt3 and Spt8 Are Involved in the Subtelomere Silencing Boundary

We investigated boundary regions within 20 kb of the telomere using the one-sample *t*-test. In the *spt3Δ* strain, the expression of genes located at subtelomere regions of chromosome III right (IIIR), IV left (IVL), IVR, VIIIL, VIIIR, XVL, and XVIR were significantly downregulated. In particular, genes on the chromosome III right telomere region (Chr IIIR) showed significant variations in *spt3Δ*, which was also the case for the *spt8Δ* strain. Therefore, we focused on Chr IIIR.

**Figure 3:**
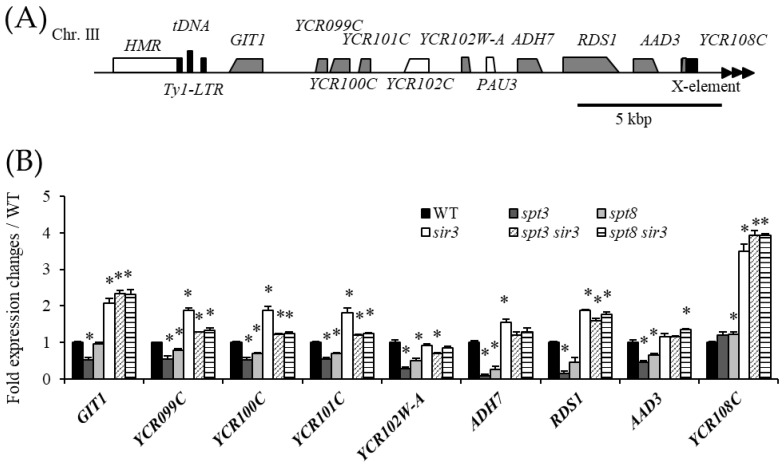
Spt3 and Spt8 are involved in boundary formation. (**A**) Gene map of Chr IIIR. Grey genes were measured gene expression. (**B**) Transcription level at Chr IIIR genes in the *spt3Δ* strain and *spt8Δ* strain, and in the *sir3Δ* strain by RT-qPCR. Asterisks mean *p* < 0.05 compared with WT (Student’s *t*-test). Error bars show standard errors calculated from three independent experiments.

To confirm the microarray results, we measured gene expression at the Chr IIIR by RT-qPCR (Figure 3). In Figure 3A, the genes analyzed are indicated in gray. Chr IIIR genes were mostly downregulated in the *spt3Δ* strain (Figure 3B). In the *spt3Δ* strain, expression of all genes was downregulated at the Chr IIIR except for *YCR108C*. Among them, the expression levels of *AAD3*, *RDS1*, *ADH7*, and *YCR102W-A* were less than 50% of the WT. Similar results were observed for *spt8Δ*, but the effect was not as great as in *spt3Δ*. There were no transcriptional changes in *YCR108C* expression in both the *spt3Δ* strain and *spt8Δ* strain. Because *YCR108C* may always be silenced by Sir2/3/4 proteins or is intrinsically not transcribed, *YCR108C* expression showed no changes.

To determine whether this wide range of repression was attributed to SAGA complex regulation or to silencing spread, *SIR3* was deleted from the *spt3Δ* or *spt8Δ* strains (Figure 3B). The expression of Chr IIIR genes in double mutant strains was comparable to that in the *sir3Δ* strain. This result indicated that repression of Chr IIIR genes was caused by silencing spread rather than SAGA complex regulation.

### 3.3. Interaction between Spt3 and TBPs Is Important for Telomere Silencing Boundary Formation

The results suggested that Spt3 and Spt8 contributed to the formation of the Chr IIIR silencing boundary. However, the specific function required for boundary formation remained unclear. It is remarkable that the patterns of gene expression in *spt3Δ* and *spt8Δ* strains were distinctly different between *YCR108C*, which is located near the telomere, and *AAD3*, which is located right next to *YCR108C* (Figure 3B).

**Figure 4:**
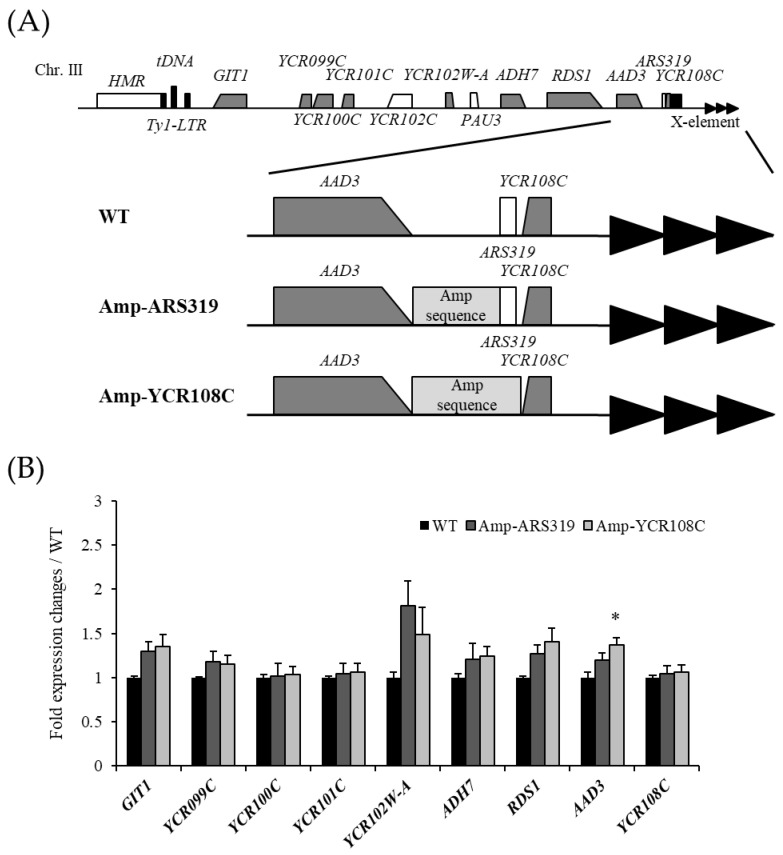
The boundary formation is DNA sequence-independent at the Chr III right telomere. (**A**) Gene map of Chr IIIR in Amp-ARS319 and Amp-YCR108C strains. (**B**) Expression level of Chr IIIR genes in Amp-ARS319 and Amp-YCR108C strains by RT-qPCR. Asterisks mean *p* < 0.05 compared with WT (Student’s *t*-test). Error bars show standard errors calculated from three independent experiments.

Therefore, we hypothesized that a DNA sequence-dependent silencing boundary exists between *YCR108C* and *AAD3*. To test this hypothesis, we generated a yeast strain in which the region between *AAD3* and *YCR108C* was replaced by an ampicillin resistance (amp^R^) gene sequence, which is normally not present in yeast. Considering the possibility that the ARS319 sequence between *AAD3* and *YCR108C* may affect boundary formation, we generated the Amp-ARS319 strain, which replaced the region from *AAD3* to ARS319, and the Amp-YCR108C strain, which replaced the region from *AAD3* to *YCR108C* (Figure 4A), and performed RT-qPCR at the Chr IIIR. Contrary to expectations, insertion of the amp^R^ gene sequence into the intergenic region (Amp-ARS319 and Amp-YCR108C) did not affect gene expression within the Chr IIIR, indicating that the regulation of the silencing region by boundary formation is independent of the DNA sequence between *AAD3* and *YCR108C* at Chr IIIR (Figure 4B).

Based on these results and the known interaction between Spt3/8 and TBP [29,30,31], we predicted that transcription of the genes at Chr IIIR would be activated by SAGA-TBP, which would suppress the spread of the silencing region from the telomere. To elucidate the effects of the SAGA-TBP interaction on transcriptional regulation at Chr IIIR, we first focused on Spt3, a TBP binding partner within the SAGA complex. The mutants of Spt8, another TBP interaction partner that is a component of SAGA complex, were not used because *spt8Δ* had a weaker effect on gene expression changes than *spt3Δ*.

**Figure 5:**
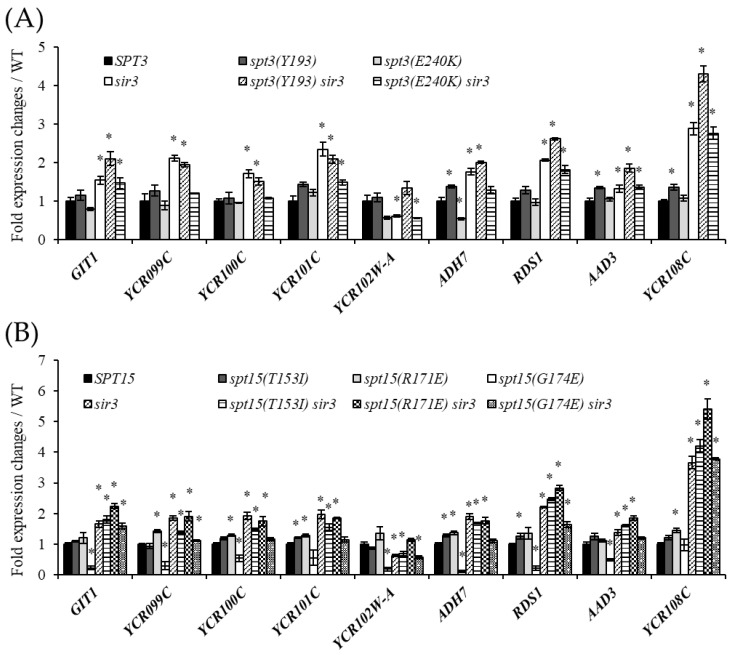
Interaction between Spt3 and TBP is important for boundary formation. (**A**,**B**) Transcription level at Chr IIIR genes in *spt3* mutant strains and *sir3Δ* strains (**A**) and in *spt15* mutant strains and *sir3Δ* strains by RT-qPCR (**B**). Asterisks represent *p* < 0.05 compared with the WT (Student’s *t*-test). Error bars show standard errors calculated from three independent experiments.

We investigated the effect of two previously identified mutations in the SPT3 gene that affect the interaction of Spt3 with TBP on gene transcript levels at Chr IIIR. The first Spt3 (Y193C) mutant strain (*spt3(Y193C)*) produces Spt3 with a Y193C amino acid residue substitution that attenuates the interaction with TBP. Conversely, the second Spt3 (E240K) mutant strain (*spt3(E240K)*) has Spt3 with an E240K substitution resulting in a stronger interaction with TBP and reduced TBP recruitment to the TATA box [16,28,49]. Both of these substitutions affect the SAGA-TBP interaction and, like *spt3Δ*, are assumed to disrupt the boundary and repress gene expression at Chr IIIR. Contrary to expectations, *spt3(Y193C)* did not show a decrease in gene expression (Figure 5A). In addition, deletion of the *SIR3* gene in the *spt3(Y193C)* strain (*spt3*(*Y193C*) *sir3Δ*) resulted in slightly higher expression levels of some genes than in the *sir3Δ* strain. Transcription of some genes was downregulated in *spt3(E240K)*, as well as gene transcription levels in the *spt3(E240K) sir3Δ*, in contrast to *spt3(Y193C)*. These results indicated that in some genes at Chr IIIR, the effects of Y193C and the E240K substitutions in Spt3 were dependent on the presence of Sir3 protein.

Next, we analyzed the effects of TBP mutations on the transcription level of genes at Chr IIIR. Three TBP (Spt15) amino acid residue substitution mutants, T153I, R171E, and G174E, have been reported to weaken TBP recruitment to the TATA box, interaction with Spt8, and interaction with Spt3 [28,35,50,51,52]. We used TBP mutant strains (*spt15(T153I), spt15(R171E), spt15 (G174E)*) and analyzed the transcription level of genes at Chr IIIR to determine whether the recruitment of TBP by SAGA or the transfer of TBP to the TATA box via Spt3 or Spt8 affects boundary formation. No transcriptional repression was observed for genes at Chr IIIR in the *spt15(T153I)* strain and *spt15(R171E)* strain, showing a similar trend to WT. The *spt15(T153I) sir3Δ* strain and *spt15(R171E) sir3Δ* strain also showed similar results to the *sir3Δ*, so TBP recruitment and interaction with Spt8 did not critical affect boundary formation at Chr IIIR. However, Chr IIIR genes were significantly repressed in the *spt15 (G174E)* mutant. Furthermore, we confirmed that gene expression at Chr IIIR in *spt15(G174E)* was restored by *sir3Δ* (*spt15(G174E) sir3Δ*). These results were similar to those obtained using the *spt3Δ* strain. This indicated that proper interaction between TBPs and Spt3 is critical for boundary formation.

### 3.4. The SAGA Complex Is Required for Boundary Formation

**Figure 6:**
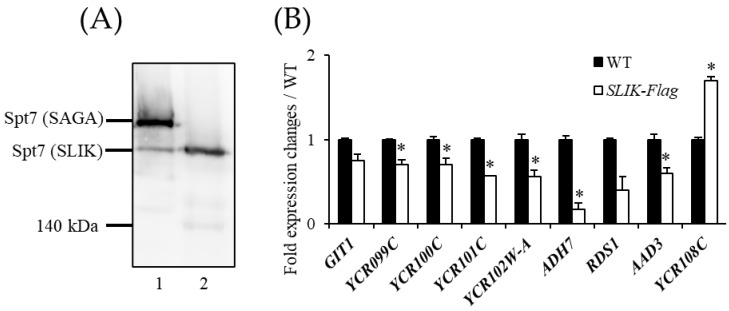
The SLIK complex is not sufficient for boundary formation. (**A**) Western blot analysis of protein extracts from strains producing Flag-Spt7 (lane 1) or SLIK-specific Spt7-Flag (lane 2). A 6% acrylamide gel was used for protein fractionation. Flag-tag was detected by an anti-Flag antibody. (**B**) Expression level of Chr III genes in the SLIK-Flag strain by RT-qPCR. Asterisks represent *p* < 0.05 compared with the WT (Student’s *t*-test). Error bars show standard errors calculated from three independent experiments.

The results described above suggested that interaction between the SAGA complex and TBPs is important for Chr IIIR boundary formation. The SLIK complex, which is similar to the SAGA complex, does not contain Spt8, whereas it does contain a truncated form of Spt7 (C-terminal truncated resulting in a protein of 1–1141 amino acid residues). However, it is unclear when SAGA and SLIK are used differently in cells. Therefore, we constructed a yeast strain in which only SLIK complexes were present and measured transcriptional expression of Chr IIIR genes. To confirm presence of truncated Spt7 (SLIK-specific), a 3xFlag-tag was added to the C-terminus (SLIK-Flag strain). We extracted and fractionated proteins from the SLIK-Flag strain and the strain expressing Spt7 with a 3xFlag-tag at the C-terminus (Flag-Spt7 strain) as a control for forming the SAGA and SLIK complexes, and detected bands of the SAGA and SLIK complexes by Western blotting using an anti-Flag antibody (Figure 6A). A band was detected in the SLIK-Flag strain at the same position as the second band of Flag-Spt7 (SLIK complex), confirming the formation of the SLIK complex in the SLIK-Flag strain. Assessment of the transcriptional expression of Chr IIIR genes with the SLIK strain (Figure 6B) showed that *AAD3*, *RDS1*, *ADH7*, *YCR102W-A*, and *YCR101C* were downregulated to approximately 20–60%, and *YCR100C*, *YCR099C*, and *GIT1* expression levels were decreased to approximately 70%. This expression pattern was similar to that of *spt8Δ* strains (Figure 3B). The above results suggested that Spt3 functions in the boundary formation by SAGA-TBP at Chr IIIR, and Spt8 is predicted to assist in the interaction between Spt3 and TBP by maintaining the structure of SAGA.

### 3.5. Spt3 and TBP Mutations Lead to the Spread of the Silencing Region

**Figure 7:**
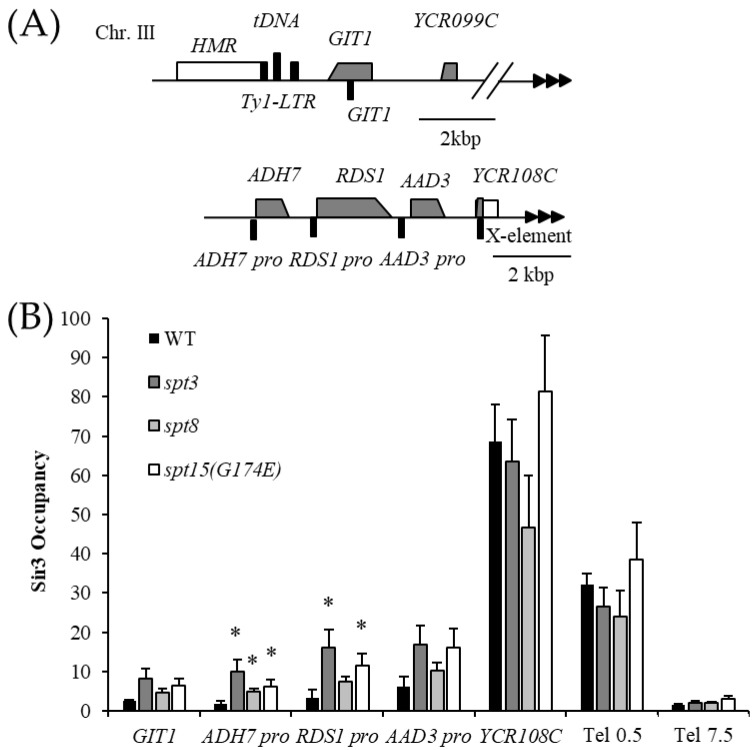
Silencing region was spread in *spt3Δ*, *spt8Δ*, and *spt15* mutants. (**A**) Primer position for the ChIP assay. Black bars mean primer positions. (**B**) Occupancy levels of Sir3 in WT, *spt3Δ*, *spt8Δ,* and *spt15(G174E)* strain. Asterisks mean *p* < 0.15 compared with WT (Student’s *t*-test). Error bars show standard errors calculated from three independent experiments.

The results suggested spreading of the silencing region at Chr IIIR in *spt3*, *spt8*, and *spt15* mutants. To determine whether the transcriptional repression was attributed to silencing region spread by Sir2/3/4 complex, we performed chromatin immunoprecipitation (ChIP) analysis targeting Sir3 in the *spt3Δ*, *spt8Δ*, and *spt15(G174E)* strains at Chr IIIR (Figure 7A). Tel 0.5 and Tel 7.5 served as positive and negative controls, respectively. In all strains, Sir3 level was not significantly changed at *YCR108C*, which is the intrinsically silenced gene. The Sir3 level was significantly increased at the *RDS1* and *ADH7* promoter regions (Figure 7B). This results strongly suggested that Spt3 and Spt8 are involved in the formation of the Chr IIIR silencing boundary.

## 4. Discussion

In this study, we focused on the SAGA complex as one of the boundary-forming factors involved in the silencing of Chr IIIR by analyzing the contribution of its components, Spt3 and Spt8, to boundary formation. We found that boundary formation at Chr IIIR was regulated by the interaction between TBP and Spt3 and/or Spt8, which are components of the SAGA complex. However, although our results suggested that the boundary formed by SAGA-TBP was located between *AAD3* and *YCR108C* (Figure 3B), replacing the sequence in this intergenic region with an amp^R^ sequence did not affect the transcription of genes at Chr IIIR, suggesting that boundary formation at Chr IIIR is not dependent on the DNA sequence between *AAD3* and *YCR108C* (Figure 4B). However, gene expression levels throughout Chr IIIR were decreased not only in the *spt3Δ* and the *spt8Δ*, which are involved in the interaction with TBP, which is important for transcription, but also in the *spt15(G174E)* mutant strain, which has a weakened interaction with Spt3, and the SLIK-Flag strain, which lacks Spt8 (Figure 3B, Figure 5B and Figure 6B). We attribute this to a decrease in the amount of TBP recruited to the TATA box, which resulted in decreased transcription and failure of TBP to function as a boundary. In fact, transcription was restored in *sir3Δ* of these mutants. We propose that SAGA acts at *ADH7* to create a transcription-friendly environment for the two adjacent telomere-proximal genes *RDS1* and *AAD3*. This “synergy in transcriptional activity” creates a boundary at *AAD3* that blocks the spread of the silencing region from the telomere. Indeed, among the genes in this region, *ADH7* has been reported to have a TATA sequence that is more SAGA-dominant than TFIID [53,54], and in our experiments, the transcription of *ADH7* was reduced in each mutant strain (*spt3Δ*, *spt8Δ*, and *spt15(G174E)*) (Figure 3B and Figure 5B). Furthermore, the transcript levels of *RDS1* and *AAD3* were higher than those of *ADH7*, suggesting that the transcription of these genes functions synergistically as a boundary. However, our results do not prove that SAGA complex directly affects this subtelomere region. Therefore, it is possible that factors that truly form the boundary of the Chr IIIR are under the control of SAGA-TBP. This is also the case because the SAGA complex regulates the expression of stress response genes, and it is assumed that genes induced in response to specific stresses are responsible for the regulation of boundary formation at Chr IIIR. It also cannot be ruled out that the downregulation of gene expressions at Chr IIIR by mutant strains of SAGA complex and TBP may be the result in the spreading of the silencing region due to the indirect upregulation of *SIR* genes by the mutation.

The *spt8Δ* strain showed gene repression at Chr IIIR and other subtelomeres (VIIR, IVL, and IXL) (Figure 3). The SAGA and SLIK complexes regulate stress response genes, and subtelomere regions contain many stress response genes [47,48]. Normally, the SAGA complex is more abundant than the SLIK complex; however, the abundance of the SAGA complex is decreased under *spt8Δ*. Although SAGA/SLIK complexes regulate stress response genes, subtelomere genes were repressed in the *spt8Δ* strain (Figure 3). This result appears contradictory because upregulation of the SLIK complex in response to stress decreases the stability of the silencing boundary. Furthermore, TFIID or SAGA complex dependency is not detected at boundary collapsed regions [32]. Further analysis is needed to resolve the discrepancy.

The boundary is maintained when a complete SAGA complex is formed. However, the interaction between SLIK and TBPs is weakened when the complete SAGA complex is not formed, as in the SLIK-Flag strain, *spt8Δ* strain, or *spt3Δ* strain. Therefore, it is possible that the SLIK complex performs part of the SAGA complex function through the interaction. We hypothesized that this was a cause of the silencing boundary collapse in the *spt8Δ* strain. Indeed, the boundary was destabilized in the SLIK-Flag strain (Figure 6). In addition, there are two possible reasons for the smaller effects on the boundary in the *spt8Δ* or SLIK-Flag strain than in the *spt3Δ* strain. First, the interaction of TBPs with Spt8 was weaker than that with Spt3. Second, there are SAGA complexes lacking Spt8 (not complete SAGA) in a certain ratio. Actually, the *YCR108C* expression level was increased. Possibly, this is an unnatural effect specific to the SLIK-Flag strain, since the *spt8Δ* strain has an incomplete SAGA complex lacking Spt8, whereas the SLIK-Flag strain cannot make the SAGA complex at all. Therefore, *spt8* deletion had a smaller effect on boundary formation as *spt3Δ*.

The silencing boundary was collapsed in the *spt3Δ*, *spt8Δ*, the SLIK-Flag strain, and the *spt15(G174E)* mutant strain with a weakened interaction with Spt3, indicating that the boundary function of the SAGA complex required interaction with TBPs to a certain extent (Figure 5). However, almost no transcriptional repression was observed at Chr IIIR in *spt3* mutants compared to *spt3Δ* or *spt15(G174E)*. In particular, *spt3(E240K)* was not only identified as a suppressor of *spt15(G174E)*, it was also reported to bind TBP more strongly than the WT and reduce TBP recruitment to the TATA-box, without much effect on transcription [16,28,51]. However, *spt3(E240K)* functioned in the absence of Sir3 protein, as the *spt3(E240K) sir3Δ* strain showed lower transcript levels in some genes than the *sir3Δ* strain. Since *spt3(Y193C)* was identified as a suppressor of *spt3(E240K)*, we expected to see a decrease in the expression levels of some genes, such as *spt3Δ* and *spt15(G174E)*, but interestingly, an increase in transcript levels was observed for some genes. This result indicated that the interaction between Spt3 and TBP is weakened in *spt3(Y193C)* and that the increased recruitment of TBP to the TATA-box may have increased transcription. This effect was also stronger in the absence of Sir3 for some genes. This suggests that the interaction between SAGA and TBP may differ between open chromatin regions and silencing regions.

The SAGA complex interacts with TFIIS through Spt8. If this interaction was required for boundary function, it was presumed that *spt8Δ* or SLIK-Flag strain would destabilize the boundary, but not *spt3Δ* or *spt15* mutants. However, the boundary was collapsed not only in *spt8Δ* or SLIK-Flag strains, but also in *spt3Δ* or *spt15* mutants. This indicated that the interaction between SAGA and TFIIS is not required for boundary formation.

## Figures and Tables

**Table 1:** Yeast strain list.

Strain No.	Genotype	Source
FUY 837	*MATa his3* *Δ1 leu2* *Δ0 met15* *Δ0 ura3* *Δ0*	This study
FUY 1621	*MATa his3* *Δ1 leu2* *Δ0 met15* *Δ0 ura3* *Δ0 spt3* *Δ::KanMX*	This study
FUY 1622	*MATa his3* *Δ1 leu2* *Δ0 met15* *Δ0 ura3* *Δ0 spt8* *Δ::KanMX*	This study
FUY 1624	*MATa his3* *Δ1 leu2* *Δ0 met15* *Δ0 ura3* *Δ0 sir3* *Δ::HphMX*	This study
FUY 1657	*MATa his3* *Δ1 leu2* *Δ0 met15* *Δ0 ura3* *Δ0 spt3* *Δ::KanMX sir3* *Δ::HphMX*	This study
FUY 1658	*MATa his3* *Δ1 leu2* *Δ0 met15* *Δ0 ura3* *Δ0 spt8* *Δ::KanMX sir3* *Δ::HphMX*	This study
FUY 1662	*MATa his3* *Δ1 leu2* *Δ0 met15* *Δ0 ura3* *Δ0 SPT3::3xFlag-SPT3*	This study
FUY 1663	*MATa his3* *Δ1 leu2* *Δ0 met15* *Δ0 ura3* *Δ0 SPT7::3xFlag-SPT7*	This study
FUY 1664	*MATa his3* *Δ1 leu2* *Δ0 met15* *Δ0 ura3* *Δ0 SPT8::3xFlag-SPT8*	This study
FUY 1665	*MATa his3* *Δ1 leu2* *Δ0 met15* *Δ0 ura3* *Δ0* *TBP(SPT15)::3xFlag-TBP(SPT15)*	This study
FUY 1757	*MATa his3* *Δ1 leu2* *Δ0 met15* *Δ0 ura3* *Δ0* *spt3* *Δ::Flag-spt3(Y193C)*	This study
FUY 1758	*MATa his3* *Δ1 leu2* *Δ0 met15* *Δ0 ura3* *Δ0* *spt3* *Δ::Flag-spt3(E240K)*	This study
FUY 1759	*MATa his3* *Δ1 leu2* *Δ0 met15* *Δ0 ura3* *Δ0* *spt15* *Δ::Flag-spt15(T153I)*	This study
FUY 1760	*MATa his3* *Δ1 leu2* *Δ0 met15* *Δ0 ura3* *Δ0* *spt15* *Δ::Flag-spt15(R171E)*	This study
FUY 1761	*MATa his3* *Δ1 leu2* *Δ0 met15* *Δ0 ura3* *Δ0* *spt15* *Δ::Flag-spt15(G174E)*	This study
FUY 1762	*MATa his3* *Δ1 leu2* *Δ0 met15* *Δ0 ura3* *Δ0* *spt3* *Δ::Flag-spt3(Y193C) sir3* *Δ::HphMX*	This study
FUY 1763	*MATa his3* *Δ1 leu2* *Δ0 met15* *Δ0 ura3* *Δ0* *spt3* *Δ::Flag-spt3(E240K) sir3* *Δ::HphMX*	This study
FUY 1764	*MATa his3* *Δ1 leu2* *Δ0 met15* *Δ0 ura3* *Δ0* *spt15* *Δ::Flag-spt15(T153I) sir3* *Δ::HphMX*	This study
FUY 1765	*MATa his3* *Δ1 leu2* *Δ0 met15* *Δ0 ura3* *Δ0* *spt15* *Δ::Flag-spt15(R171E) sir3* *Δ::HphMX*	This study
FUY 1766	*MATa his3* *Δ1 leu2* *Δ0 met15* *Δ0 ura3* *Δ0* *spt15* *Δ::Flag-spt15(G174E) sir3* *Δ::HphMX*	This study
FUY 1840	*MATa his3* *Δ1 leu2* *Δ0 met15* *Δ0 ura3* *Δ0* *spt7* *Δ::SPT7(1-1141)-3xFlag*	This study

**Table 2:** Primer list for qPCR.

Primer No.	Sequence
832-GIT1/YCR098C-F	CCAAAAGAGGTGGTATCCTGGTT
833-GIT1/YCR098C-Rv	TGGACCACCGAAGGCTAGTG
834-YCR099C-F	AATGCAAAAAGCCCATGGAA
835-YCR099C-Rv	CTCTCCCTCAGGATTTTTTCACA
837-YCR100C-C	GGGCCACCCTCCATGTTAG
838-YCR101C-F	TGGGAAACGGTCAAAGAAATTG
839-YCR101C-Rv	CCATGGAAAGGATCAACAGTAAATC
842-YCR102W-A-F	GAGGAAAAGTTTGGAAGAACAAAAA
843-YCR102W-A-Rv	CTCCCCGTAAAGAATGCTTGAT
852-AAD3/YCR107W-F	GCGCCTCCGAACAAACAG
853-AAD3/YCR107W-Rv	AGCAATCTTGGCCAATGCTT
854-YCR108C-F	CCATGGCCCATTCTCACTAAA
855-YCR108C-Rv	CAAGTGCCGTGCATAATGATG
1503-836-YCR100C-F	CGATCGGAAGGACCGAAAA
1707-851-RDS1/YCR106W-2Rv	GGACATAGCGGTATTGGCTTTT
1708-oki264 GIT1-F	TGGATGTGCGTACGACCAAT
1709-oki265 GIT1-Rv	ACCTGGTCCAGCATTACCTAACA
1719-847-ADH7/YCR105W-Rv	TCTCCGCTTTCCATCCTTGT
2882-846-ADH7/YCR105W-F	AAACTTCCGATCAGCGAAGAAG
2883-850-RDS1/YCR106W-2F	GCCAGATGGAGGATGCAGTT

**Table 3:** Primer list for ChIP.

Primer No.	Sequence
854-YCR108C-F	CCATGGCCCATTCTCACTAAA
855-YCR108C-Rv	CAAGTGCCGTGCATAATGATG
1708-oki264 GIT1-F	TGGATGTGCGTACGACCAAT
1709-oki265 GIT1-Rv	ACCTGGTCCAGCATTACCTAACA
3055-AAD3(-1)-R	TTTAAGCACGATGGATATGCTTC
3056-AAD3(-82)-F	CTTTTGCTGGTTTCGATGATG
3061-RDS1(-1)-R	CAGAGCATTTCAGCAGCCAA
3062-RDS1(-93)-F	GACATCATTACTAATAATGTTACTC
4827-ADH7 pro 1 F	CGGCCGCATAATAAAATGGA
4828-ADH7 pro 1 Rv	TCTACTCAGAGTTTTGGTGCTCAATT
4850-tel(VI)0.5(F)	CCTTTTTTGATATAACTGTCGGAGAGT
4851-tel(VI)0.5(Rv)	TCCGAACGCTATTCCAGAAAGT
4852-tel(VI)7.5(F)	TGTAGACTTCCCACTGTATTTGAATGA
4853-tel(VI)7.5(Rv)	CGTGAAAGTTCAGCGCAACA

## Data Availability

Experimental data of microarray from this study are deposited in the NCBI Gene Expression Omnibus database. This data (accession number GSE220290) can be found here: https://www.ncbi.nlm.nih.gov/geo/query/acc.cgi?acc=GSE220290 (accessed on 20 February 2023).
